# Evaluation of Capillary Blood Gases in Medical Personnel Caring for Patients Isolated Due to SARS-CoV-2 in Intensive Care Units before and after Using Enhanced Filtration Masks: A Prospective Cohort Study

**DOI:** 10.3390/ijerph18189425

**Published:** 2021-09-07

**Authors:** Wioletta Mędrzycka-Dąbrowska, Daniel Ślęzak, Marlena Robakowska, Przemysław Żuratyński, Kamil Krzyżanowski, Anna Małecka-Dubiela, Sebastian Dąbrowski, Katarzyna Zorena, Katarzyna Lewandowska, Dorota Ozga, Karina Chmielarz, Paulina Buca, Lucyna Tomaszek

**Affiliations:** 1 Department of Anaesthesiology Nursing and Intensive Care, Faculty of Health Sciences, Medical University of Gdańsk, Dębinki 7, 80-211 Gdańsk, Poland; kalewandowska@gumed.edu.pl; 2 Department of Medical Rescue, Faculty of Health Sciences, Medical University of Gdańsk, Dębinki 7, 80-211 Gdańsk, Poland; daniel.slezak@gumed.edu.pl (D.Ś.); przemyslaw.zuratynski@gumed.edu.pl (P.Ż.); kamil.krzyzanowski@gumed.edu.pl (K.K.); sebastian.dabrowski@gumed.edu.pl (S.D.); 3 Department of Public Health & Social Medicine, Faculty of Health Sciences, Medical University of Gdańsk, 80-211 Gdańsk, Poland; marlena.robakowska@gumed.edu.pl; 4 Department of Internal and Pediatric Nursing, Faculty of Health Sciences, Medical University of Gdańsk, Dębinki 7, 80-211 Gdańsk, Poland; anna.malecka-dubiela@gumed.edu.pl; 5 Department of Immunobiology and Environment Microbiology, Faculty of Health Sciences, Medical University of Gdańsk, Dębinki 7, 80-211 Gdańsk, Poland; katarzyna.zorena@gumed.edu.pl; 6 Institute of Health Sciences, College of Medical Sciences of the University of Rzeszow, St. Warzywna1A, 35-310 Rzeszow, Poland; gdozga@poczta.fm; 7 Department of Laboratory Diagnostics, Nicolaus Copernicus University of Toruń, Collegium Medicum im. L. Rydygier in Bydgoszcz, Jagiellońska 13/15, 85-067 Bydgoszcz, Poland; karina.chmielarz@gmail.com; 8 Division of Hyperbaric Medicine & Maritime Rescue—National Centre for Hyperbaric Medicine, Faculty of Health Sciences, Medical University of Gdańsk, Powstania Styczniowego 9b, 81-519 Gdynia, Poland; paulina.buca@gumed.edu.pl; 9 Institute of Tuberculosis and Lung Diseases, Rabka-Zdrój Branch, ul. Prof. Jana Rudnika 3B, 34-700 Rabka-Zdrój, Poland; ltomaszek@igrabka.edu.pl

**Keywords:** masks, pulmonary ventilation, SARS-CoV-2, ICU, human hydration

## Abstract

The dynamically changing epidemiological situation caused by the SARS-CoV-2 virus is associated with the increased burden and fatigue of medical personnel. The aim of the study was to evaluate: (1) oxygen and carbon dioxide blood pressure and saturation levels in medical personnel caring for patients isolated due to SARS-CoV-2 in ICUs; (2) adverse symptoms reported by medical personnel after leaving the isolation zone. Design: A Prospective Cohort Study. Methods: The project was implemented in the first quarter of 2021. Medical personnel working with patients isolated due to SARS-CoV-2 in the ICU of three hospitals were eligible for the study. The participants of the study were subjected to two analyses of capillary blood by a laboratory diagnostician. Results: In the studied group of medical personnel (n = 110) using FFP2/FFP3 masks, no significant differences (*p* > 0.05) were found between the parameters of geometric examination performed before and after leaving the isolation ward of the hospital. After working in the isolation ward, nurses reported malaise (somnolence, fatigue, sweating, dizziness) more often than paramedics (44% vs. 9%; *p* = 0.00002). The risk of ill-being in nurses was approximately nine times higher than in paramedics (OR = 8.6; Cl 95%: 2.7 to 26.8) and increased with the age of the subjects (OR = 1.05; Cl 95%: 1.01 to 1.08). Conclusion: FFP2/FFP3 filter masks did not worsen blood oxygenation in medical staff caring for patients isolated due to SARS-CoV-2 in the ICU. The presence of subjective symptoms such as fatigue may be due to lack of adequate hydration.

## 1. Introduction

The dynamically changing epidemiological situation caused by the SARS-CoV-2 virus is associated with the increased workload and fatigue of medical personnel. Prolonged use of personal protective equipment (PPE) may contribute to this. Particularly vulnerable groups, due to the time they must spend in PPE, are nurses working in the intensive care unit for patients infected with SARS-CoV-2 and the paramedics who support them [1,2]. Transmission of acute respiratory infections occurs mainly by contact, droplet, and probably airborne routes. Therefore, the use of a mask with enhanced filtration efficiency, a visor, goggles, a barrier apron, or a diagnostic suit and gloves should be considered appropriate personal protective equipment during routine care of a patient with a highly infectious acute respiratory infection [3,4]. Concerns have been raised among medical personnel regarding the adverse effects of the prolonged use of masks with enhanced filtration efficiency on pulmonary gas exchange [5,6]. The mask, by generating resistance, increases the work of breathing, which creates the possibility of partial reflux due to decreased supply of fresh gases from the environment and, consequently, the risk of hypoxemia and hypercapnia. To ensure proper homeostasis of the body, chemoreceptors in the respiratory center activate compensatory mechanisms to maintain acid-base balance and blood oxygenation within normal limits. This induces increased effort in breathing and hyperventilation. The use of protective masks with normal permeability allows normocapnia and normal blood oxygenation to be maintained. However, long-term mask wearing may be associated with adverse effects, described in the literature as headache, cognitive dysfunction, or skin lesions. Most often the symptoms resolve spontaneously after the mask is removed; however, there are situations where medical intervention is necessary [7,8].

### Aim

The purpose of this study was: (1) to evaluate the levels of oxygen and carbon dioxide pressure and saturation in arterialized capillary blood collected from medical personnel caring for patients isolated due to SARS-CoV-2 in the ICU; (2) to evaluate the adverse symptoms reported by medical personnel after leaving the isolation zone.

## 2. Methods

### 2.1. Study Design, Setting, Ethical Considerations

This was a prospective cohort study that included 110 medical staff caring for patients isolated due to SARS-CoV-2 in the ICU of three hospitals in Poland. The study was approved by the Bioethics Committee of University of Rzeszow. The authors followed the guidelines of the Declaration of Helsinki (World Medical Association, 2013) and STROBE (Strengthening the Reporting of Observational Studies in Epidemiology) [9]. The research was conducted in the first quarter of 2021. The necessary staffing of medical personnel in the care of patients isolated and requiring mechanical ventilation due to SARS-Co infection in the ICU is regulated by the Position Statement of the Team of Consultants in Anesthesia and Intensive Care Nursing in agreement with the Main Board of the Polish Society of Anesthesia and Intensive Care Nurses [10].

### 2.2. Participants Clinical

The study involved nurses and paramedics who: gave informed consent to participate in the study; worked in a hot (contaminated) zone for a minimum of 2 h [3]; worked in a 4-h shift system; were protected by a properly selected mask with an FFP2 visor/FFP3 +, equipped with personal protective equipment, depending on the degree of risk, that did not burden the respiratory system.

The study did not include nurses and paramedics who: did not agree to participate in the study; worked in the cold zone; worked shifts for less than 1 h; used a surgical mask; had a history of respiratory diseases.

### 2.3. Research Procedure

Two capillary blood samples were taken from the subjects for Capilar Blood Gases Test. The first blood sampling was performed before securing the medical personnel with personal protective equipment. The second blood sampling was performed immediately after completion of the isolation care [2]. Each time, blood was collected by individuals from the research team, from the fingertip after warming the puncture site (massaging). A Radiometer ABL90 Flex Plus critical parameters analyzer was used for the study. The reference norms were described as: pH, 7.35–7.45; PaO_2_, 75–100 mmHg; PaCO_2_, 35–45 mmHg; HCO_3_^−^, 22–28 mmol/l; O_2_sat, 70–95%; BE +/− 2.5 mmol/L [11]. Participants who consented to participate in the study were read the Informed Consent Form. Participation in the study was voluntary. Each participant was asked open-ended questions regarding subjective well-being before and after being released from isolation.

### 2.4. Data Collection

Data collection included: age, body weight, body height, gender, profession, co-morbidities, time of working in an isolation ward, number of times entering the isolation ward, type of medical protective mask, adverse events connected with work in an isolation ward.

### 2.5. Outcomes

The primary outcomes included the assessment of oxygen and carbon dioxide pressure and saturation levels in arterialized capillary blood. The secondary outcomes were the assessment of adverse symptoms reported by medical staff after leaving the isolation zone.

### 2.6. Statistics

The minimum size of the sample was determined a priori with G*Power (version 3.1.9.4, Germany). Given an effect size of 0.3333 with a minimum of 90% power, the minimum number of participants was calculated as 97 with a 5% type 1 error.

Continuous quantitative variables were characterized as medians and quartiles, while qualitative variables were presented as counts of each level with corresponding percentages. Quantitative variables were compared between dependent groups (Before vs. After) by Wilcoxon probability tests (Wilcoxon tests) due to the lack of normal distribution of these variables in both groups. Normal distribution of quantitative variables was determined by the Shapiro–Wilk test. The chi-square test or Fisher’s test was used to compare qualitative variables between the study groups. Effects of age, gender, body weight, body height, smoking, time of working in an isolation ward, and profession on the physical symptoms (expressed as a dichotomous variable: no and yes) were tested in a univariate logistic regression model (B, regression coefficient; SE, standard error; CI, confidence interval; OR, odds ratio). The *p* values being below 5% were taken as statistically significant for two-sided tests. Statistical calculations were performed in STATISTICA v.13 (TIBCO Software Inc. (2017), Kraków, Poland).

## 3. Results

Sixty-three (57%) nurses and 47 (43%) paramedics were enrolled in the study. The median age of the subjects was 28 years, median height was 170 cm, and median weight was 70 kg. Thirty-eight percent of the subjects reported that they smoked cigarettes, while 4% of the subjects reported having a cardiovascular disease. Capillary blood analysis was performed in 62% of the subjects after one entry into the isolation zone, in 31% of the subjects after two entries, in 6% of the subjects after three entries, and in only one subject after four entries into the isolation zone. The median time spent in isolation was 240 min. Of note, during the entire observation period there was a significant difference between nurses and paramedics in terms of their time of working in an isolation ward (median 240 (210; 360) vs. 180 (180; 240); Z = 3.83; *p* = 0.0001). All subjects were protected with PPE, with 29% wearing a mask with an FFP2 filter, 27% of subjects wearing a half-face mask with an FFP3 filter, and 44% of subjects wearing a full-face mask with an FFP3 filter. The exact characteristics of the study group are presented in Table 1.

Table 2 presents the results of gasometry performed before and after leaving the isolation zone. There were no significant differences (*p* > 0.05) between individual parameters. Medians of pO2 (75.45 mmHg vs. 76.10 mmHg), pCO2 (37.70 mmHg vs. 37.90 mmHg), and SaO2 (96.25% vs. 96.0%) were similar. Variables such as gender, occupation, smoking, type of mask used, and number of entries into the ICU (one entry vs. more than one entry) also had no effect (*p* > 0.05) on blood gas results.

**Figure 1:**
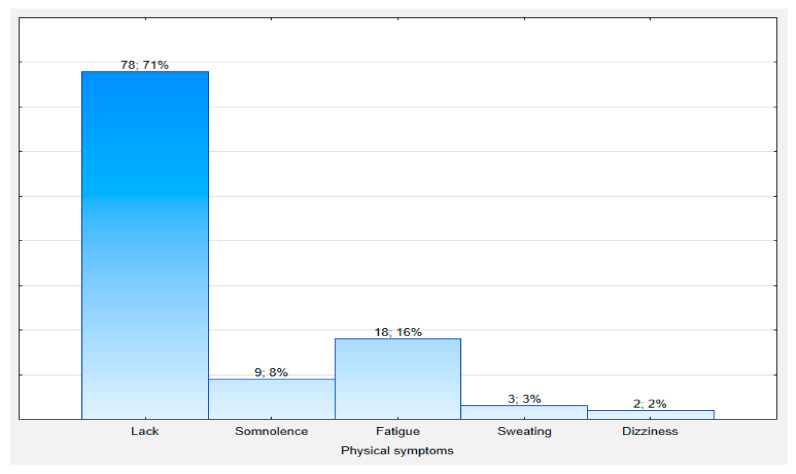
Physical symptoms reported by participants after taking care of COVID-19 patients in isolation wards.

Twenty-nine percent of subjects reported adverse symptoms after leaving the isolation zone. The most common complaints were fatigue and drowsiness (Figure 1). Nurses were more likely to report adverse symptoms than paramedics—the difference was statistically significant (*p* = 0.00002). In univariate logistic regression analysis, the risk of malaise in nurses after exiting the isolation zone was approximately nine times higher than in paramedics (OR = 8.6; Cl 95%: 2.7 to 26.8) (Table 3) and increased with age (OR = 1.05; Cl 95%: 1.01 to 1.08)—odd ratio indicates that the odds for an event increase 1.05 times when the value of the age is increased by 1 year (Table 4). Nurses were significantly older than paramedics: median 41 years (26; 50) vs. 25 years (23; 35); Z = 4.31; *p* = 0.000016.

## 4. Discussion

The results of this study suggest that FFP2/FFP3 filter masks have no effect on blood oxygenation in medical personnel caring for patients isolated due to SARS-CoV-2 in the ICU. The most common adverse reactions reported by medical personnel after leaving the isolation zone were fatigue and drowsiness. The frequency of adverse symptoms depended on age and occupation, with nurses more likely to report adverse symptoms than paramedics. One must bear in mind that paramedics in Poland, though fully qualified to provide diagnostic, treatment, and prophylactic services, especially in acute life-threatening situations across patients of all ages, have limited responsibilities in the ICU settings. Due to legal constraints, they are allowed to perform various tasks only with direct supervision of registered nurses as far as the intensive care environment is concerned. Based on current scientific evidence, the wearing of PPE, including face shields or goggles, face masks, barrier aprons or coveralls, and gloves is recommended when caring for patients with COVID-19. The use of masks is thought to reduce the spread of SARS-CoV-2 but is also a frequently cited concern for inadequate gas exchange [11]. In our study, no changes were observed in oxygen and carbon dioxide pressure and saturation levels in the capillary blood of medical personnel caring for patients isolated due to SARS-CoV-2 in the ICU, which may indicate that FFP2/FFP3 filtered masks do not significantly affect the gas exchange. A study by Shein et al. also attempted to answer the question of whether cloth or surgical masks impair oxygenation or ventilation. In the study, heart rate (HR), transcutaneous carbon dioxide (CO_2_) pressure, and oxygen level (SpO_2_) were measured at the end of six 10-min phases: sitting quietly and walking briskly without a mask, sitting quietly and walking briskly with a fabric mask, and sitting quietly and walking briskly with a surgical mask. An expected and intended increase in heart rate was observed when walking without a mask compared to sitting without a mask, while no effect on ventilation was observed [12]. Similar results were obtained in a study by Samannan et al., where researchers analyzed whether abnormalities in gas exchange occur during surgical mask use in subjects with and without pulmonary impairment. Pulse oximetry was used in the study. No changes in end-expiratory CO_2_ concentration and oxygen saturation were observed [13]. Studies by Roberge et al. [6,14] showed some increase in breathing resistance while wearing a mask, but it was not large enough to have any significant clinical implications. In a study by İpek et al., an attempt was made to find the cause of pain and headaches in personnel wearing N95 masks. Healthcare workers first put on a surgical mask for at least 1 h and a maximum of 4 h, this process was then repeated on another day with the same workers wearing N95 masks. After removing the mask, capillary blood gases were taken and a questionnaire was given. In this study, it was quantitatively shown that the participants’ symptoms were due to respiratory alkalosis and hypocarbia [15]. The analysis of our study revealed that 1/3 of medical personnel had clearly perceived subjective symptoms, i.e., fatigue or sleepiness. As Scheid et al. note [4], several other complaints may be reported during the prolonged use (>12 h) of masks, such as headaches, light-headedness, an increase in perceived exertion, and perceived shortness of breath. With a lack of any harmful physiological alterations in gas exchange, one can only speculate on other possible causes of such discomforts. One of the reasons might be staff dehydration. In intensive therapy units, the room temperature varies depending on the country and ranges from 16–25 °C, e.g., Australia, United Arab Emirates, and the United States recommend a temperature of 21–24 °C, whereas Great Britain recommends 18–25 °C and India 16–25 °C [16]. Long working hours in the ICU and additional protection in PPE may result in increased evaporation. Adequate hydration status, and thus body water content, is essential for a person’s physical health and mental well-being. Scientific evidence supports that dehydration as low as 2% of total body weight can result in increased visual vigilance errors, as well as decreased visual response latency and working memory. Mild dehydration due to water loss equivalent to 1.4% of body weight can result in decreased mood, reduced perception, difficulty with tasks, and a decreased ability to concentrate. Slight reduction in fluid intake is associated with increased subjective feelings of fatigue, headache, tension, anxiety, and cognitive decline, including short-term memory impairment [17,18]. In a study by El-Sharkawy et al., 36% of participants were dehydrated at the beginning of their shift and 45% were dehydrated at the end of their shift. Mean urine osmolality was significantly higher at the end of the shift compared to the beginning (720 (282) vs. 622 (297) mOs/kg, *p* = 0.031). This study highlights that a significant proportion of nurses and physicians were dehydrated at the beginning and end of their medical shift professionals’ attitudes towards the importance of hydration for health, well-being, and productivity. Respondents from Mediterranean countries, i.e., Italy, Spain, and Greece, believed that a good hydration strategy was a key aspect in counteracting dehydration, while respondents from Germany and the UK considered it less important. The study did not consider whether this was due to differences in climate or culture [19]. The limited fluid intake prior to entering the isolation zone may be due to concerns regarding frequent urination by medical staff, which will be associated with a shorter time in the isolation zone than 4 h. Further research is recommended with an eye toward proper hydration of medical personnel in the ICU during the SARS-CoV-2 outbreak.

## 5. Study Limitations

Due to technical limitations, we could not measure hematocrit and glucose levels, and there was no possibility of immediate gasometry assessment after leaving the isolation zone due to the necessity to undress in compliance with the sanitary regime. The type of activities performed during the stay in the ward was not analyzed, and each time they could significantly differ in terms of effort and intensity. Other potential factors influencing mental and physical well-being, such as food and fluid intake or sleep deprivation, were not taken into account.

## 6. Conclusions

FFP2/FFP3 filter masks did not impair blood oxygenation in medical personnel caring for patients isolated due to SARS-CoV-2 in the ICU. Adverse effects of being in the isolation zone were related to the profession and age of the medical staff.

### Implications for Practice

In the short term, maintaining adequate hydration can help prevent dehydration, which can cause headaches, dizziness, fatigue, fainting, rapid heartbeat, and other symptoms [20]. The importance of education should be emphasized, especially regarding the impact of fluid intake or quality and quantity of sleep on the mental and physical well-being of medical personnel.

## Figures and Tables

**Table 1:** Characteristics of participants (n = 110).

Variables	
Age (years)	28 [24; 43]
Body weight (kg)	70 [65; 78]
Body height (cm)	170 [164; 177]
Gender	
Female	74 (67%)
Male	36 (33%)
Profession	
Nurses	63 (57%)
Paramedics	47 (43%)
Co-morbidities	
Cardiovascular system	4 (4%)
Smoking	42 (38%)
Working in an isolation ward (minutes)	240 [180; 360]
Number of entries into isolation ward	
One	68 (62%)
Two	34 (31%)
Three	7 (6%)
Four	1 (1%)
Medical protective mask	
P2	32 (29%)
P3 half-face	30 (27%)
P3 full-face	48 (44%)

Categorical data were reported as number and percentage. Descriptive statistics are expressed as a median and upper and lower quartile.

**Table 2:** Arterial blood gas analysis of participants before and after taking care of COVID-19 patients in isolation wards.

Parameter	Before (n = 110)	After (n = 110)	Z	*p* Value
pH	7.42 (7.40; 7.43)	7.41 (7.39; 7.42)	1.29	0.19
pO_2_ (mm Hg)	75.45 (68.40; 81.70)	76.10 (70.70; 82.20)	1.44	0.15
pCO_2_ (mm Hg)	37.70 (35.40; 40.00)	37.90 (35.80; 40.50)	0.57	0.56
HCO_3_ (mmol/L)	24.10 (22.90; 25.20)	23.85 (22.40; 25.00)	0.16	0.87
SaO_2_ (%)	96.25 (94.40; 99.00)	96.00 (94.20; 98.00)	1.07	0.28
BE (mEq/L)	−0.4 (−1.60; 0.60)	−0.50 (−1.90; 0.50)	0.35	0.72

Results presented as median and upper and lower quartile. Hydrogen ion concentration (pH), Oxygen concentration (pO_2_); Carbon dioxide concentration (pCO_2_); Bicarbonate ion concentration (HCO_3_); Oxygen saturation (SaO_2_); Base excess (BE).

**Table 3:** Physical symptoms reported by participants after caring for patients with COVID-19 in isolation units and variables (n = 110).

Variables	No	Yes	χ^2^	*p* Value
Gender				
Female	54 (73%)	20 (27%)	0.46	0.49
Male	24 (67%)	12 (33%)		
Profession				
Nurses	35 (56%)	28 (44%)	18.73	0.00002
Paramedics	43 (91%)	4 (9%)		
Co-morbidities	31 (67%)	15 (33%)	0.47	0.49
Medical protective mask				
FFP2	23 (72%)	9 (28%)	0.20	0.90
FFP3 half-face	22 (73%)	8 (27%)		
FFP3 full-face	33 (69%)	15 (31%)		
Number of entries into isolation ward				
1	51 (75%)	17 (25%)	1.44	0.23
>1	27 (64%)	15 (36%)		

Data were reported as number and percentage.

**Table 4:** Univariate logistic regression analysis results assessing variables for the physical symptoms (n = 110).

Variables	B	SE (B)	Wald Test	*p*	OR (Cl 95%)
Profession nurse vs. paramedic	2.15	0.58	13.72	0.0002	8.6 (2.75 to 26.86)
Age	0.05	0.02	6.31	0.012	1.05 (1.01 to 1.08)
Body weight	0.01	0.02	0.57	0.45	1.01 (0.98 to 1.05
Body height	−0.01	0.01	1.23	0.27	0.98 (0.96 to 1.01
Gender	0.3	0.44	0.46	0.49	1.35 (057 to 3.18)
Smoking	−0.07	0.43	0.02	0.87	0.93 (0.39 to 2.19)
Time of working in an isolation ward	−0.003	0.002	1.51	0.21	0.99 (0.99 to 1.00)

B = regression coefficient; SE = Standard error; OR = Odds ratio; CI = Confidence interval.

## Data Availability

The authors declare that the data from this study are available from the authors P.Ż., K.K. and A.M.-D. upon request.
